# Subacute Stroke in a Young Female: A Case of Moyamoya Syndrome Initially Anchoring with Anxiety

**DOI:** 10.1155/2019/7919568

**Published:** 2019-12-10

**Authors:** Saira Chaughtai, Zeeshan Chaughtai, Muhammad Shaheryar Haider, Hasnan M. Ijaz, Sarah Elmedani, Mohamed Bakr, Mohammad A. Hossain, Arif Asif

**Affiliations:** Department of Medicine, Jersey Shore University Medical Center, Hackensack Meridian Health, Neptune, NJ 07753, USA

## Abstract

Moyamoya disease is an arterial disorder causing stroke in a young patient. This is a chronic condition causing progressive cerebrovascular disease due to bilateral stenosis and occlusion of the arteries around the circle of Willis, with prominent arterial collateral circulation. It was first described in Japan and subsequently reported in other Asian countries, but infrequently found in the Western world. Interestingly, there may be racial differences in the presentation and subsequent prognostication of treatment of moyamoya. It is diagnosed with classic angiographic findings of stenosis or occlusion of the circle of Willis vessels. Here, we describe a 28-year-old Caucasian female who was initially diagnosed with anxiety when she presented with symptoms of impaired concentration and fatigue. After the development of remitting slurred speech and facial droop, magnetic resonance imaging and cerebral angiogram yielded the discovery of high-grade stenosis of the origin of the left middle cerebral artery with associated thrombosis in that area. She did well after getting surgery and rehabilitation. This demonstrates a unique presentation of prominent psychiatric symptoms initially thought to be anxiety and culminated in the finding of ischemic stroke in an adult patient with moyamoya.

## 1. Introduction

Moyamoya disease (MMD) is an arterial disorder causing ischemic stroke in a young patient. This occurs primarily in the female sex in two age groups: the first decade of life and the fourth to fifth decades. Diagnosis relies on characteristic angiographic findings of bilateral stenoses of the arteries in the circle of Willis with collateral vessel formation [1]. This occlusion is located at the branching of the anterior cerebral artery and/or middle cerebral artery when they come off the internal carotid artery [1]. When it is only one-sided or associated with other medical conditions, it is termed moyamoya syndrome (MMS). These medical conditions include diseases such as Down's syndrome and sickle cell disease [1]. It is uncommon to have a case of moyamoya present with such prominent psychiatric symptoms. In this case summary, we discuss a case of a 28-year-old Caucasian woman who presented with prominent psychiatric symptoms. She was initially deemed to have anxiety, but subsequently developed remitting facial droop and slurred speech and found to have evidence of an ischemic stroke along with moyamoya syndrome on her cerebral imaging.

## 2. Case Summary

**Figure 1:**
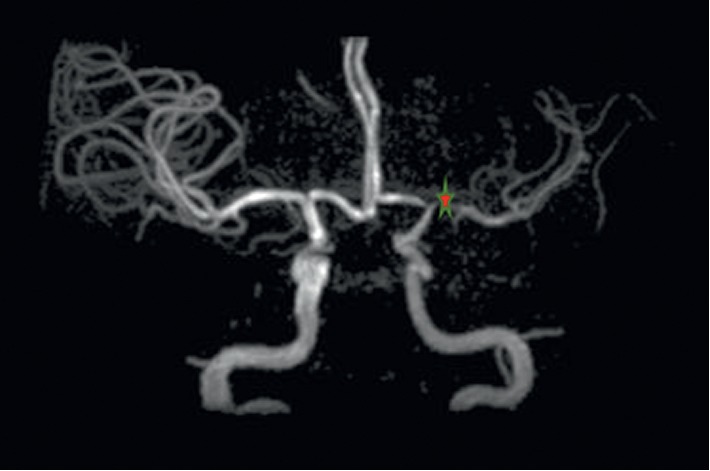
Our patient's magnetic resonance angiogram of the brain. The location of the red star shows the stenoses of the origin of the left anterior cerebral artery (A1 segment) and origin of the left middle cerebral artery (M1 segment), with associated thrombosis of the origin of the left middle cerebral artery.

A 28-year-old Caucasian female, with a history of depression and breast fibroadenoma status postresection, presented to the hospital with a one-month history of fatigue, impaired concentration, and remitting slurred speech and right-sided facial droop. Three weeks prior to this admission, she came to the emergency department with the chief complaint of a “hard time processing thoughts” and had a normal neurologic examination. She was evaluated by computed tomography (CT) of the head and discharged home as the imaging study was unremarkable and symptoms were presumed to be anxiety related as they involved a strong psychiatric component. She subsequently developed slurred speech and facial droop. For further evaluation, magnetic resonance imaging (MRI) was ordered outpatient and showed a subacute infarct of her left middle cerebral artery, prompting her primary care physician to send her to the hospital for further evaluation and management. Family history includes a maternal grandfather who died of multiple strokes at age 45. On physical examination, her heart rate was 88/min, blood pressure was 139/93, respiratory rate was 15/min, temperature was 98 degrees Fahrenheit, and pulse oxygenation was 96% on room air. On mental status exam, she displayed anxiety regarding her condition. On neurological examination, she had word-finding difficulty and difficulty in repetition; however, her comprehension was intact. She also had a right-sided central facial droop of the upper motor neuron type. Her muscle strength was normal, and sensation was intact in all extremities. Magnetic resonance angiogram (MRA) of the head and neck demonstrated stenosis of the origin of the left middle and anterior cerebral arteries with thrombosis of the former (Figure 1). Cerebral angiogram (CTA) showed high-grade stenosis of the origin of the left middle cerebral artery with associated delayed filling in that territory. A neurologist was consulted, and he started her on aspirin, clopidegrol, and statin. She remained neurologically stable during the hospital course and was discharged with outpatient neurosurgery follow-up. She eventually underwent a direct and indirect bypass of the abnormal cerebral vasculature. She also had rehabilitation for her physical symptoms and had significant clinical improvement on follow-up.

## 3. Discussion

Moyamoya is a condition that involves progressive stenosis of the arteries of the brain with concomitant collateral vessel formation [2]. The term “moyamoya” is of Japanese origin meaning a “puff of smoke,” which is the visual manifestation of the collateral circulation. Moyamoya disease is characterized by bilateral stenosis of the arteries of the circle of Willis; however, unilateral presentation is termed as moyamoya syndrome and bilateral disease eventually develops in many [2]. Moyamoya syndrome also applies to patients with certain associated conditions like Down's syndrome and sickle cell disease [2]. This condition is most commonly found in people of Asian heritage, but less so in North America [2]. Pathologic analysis shows that vessel occlusion results from a combination of hyperplasia of smooth-muscle cells and luminal thrombosis [2]. Genetic factors play a role as ten percent of Japanese cases have affected first-degree relatives. Some studies have identified mutations in proteins involved in extracellular-matrix remodeling and angiogenesis in the brain. Manifestations can be any signs of symptoms of a stroke and may cooccur with personality changes [2]. It predominantly occurs in two age groups: the first decade of life and the fourth to fifth decades of life [2]. The incidence in parts of the United States was found to be 0.086 per 100,000 [3]. Diagnosis is based upon characteristic angiogram findings involving stenosis of the distal internal carotid artery, with stenosis of the origins of the anterior and middle cerebral arteries [3]. The Suzuki grading system classifies the grade based on stenosis of the arteries with progressive collateral circulation development [3].

Treatment aims to reduce the risk of stroke and formation of collaterals by improving blood flow. However, there is no treatment that can reverse the disease [4]. Medical therapy includes antiplatelets, statins, and calcium channel blockers. However, surgery has better potential for improvement in symptoms and risk of recurrent stroke. Surgical options aim to revascularize the brain tissue and have been shown to be superior in improving the clinical course of patients with moyamoya [5]. These options include a direct and indirect approach. The indirect approach involves revascularizing the brain by placing connective tissue with vasculature on the cortical zone from which the vessels sprout [5]. The direct method involves a connection formed between a graft vessel and one of the cerebral vessels [5]. For example, the vasculature may be taken from the superficial temporal artery [5].

It is noteworthy that primarily psychiatric symptoms as the presentation of moyamoya is quite unique. Other case reports have described rare instances of patients initially presenting with anxiety, psychosis, prominent depression, and insomnia [6]. The significance of our case lies in the fact that our patient presented with prominent psychiatric symptoms of impaired concentration, anxiety, and fatigue. She was initially diagnosed as anxiety as her symptoms were associated with a prominent psychiatric component, and she was instructed to follow-up outpatient. She subsequently developed remitting facial droop and slurred speech and had an outpatient MRI done showing evidence of moyamoya. Her angiography confirmed findings, and she was able to get a direct and indirect bypass, along with rehabilitation, with ensuing improvement in symptoms. In a provider who is unaware of moyamoya, this may be labelled as a psychiatric case as her slurred speech and facial droop occurred after her initial presentation to the hospital. This highlights the importance of being aware of this diagnostic possibility in patients who we may label as having a psychiatric condition.

Since her facial droop and slurred speech were remitting, it is also easy for the provider to miss the fact that it was a stroke. Maheswari et al. mentioned in their case report that their patient had remitting symptoms of hemichorea and hemiparesis which was finally diagnosed after 3 months as being moyamoya [7]. So, it is important to know symptoms may remit and relapse.

Also, of note, most studies have involved the East Asian population; however, emerging evidence suggests that there may be a differing clinical presentation of moyamoya in other races [8]. It is noted that Asian patients are more likely to present with hemorrhage rather than ischemia [8]. Studies also suggest that non-Asian patients may have better outcomes after surgery [8]. Our Caucasian patient did have ischemia and did improve after surgery with no recurrence of stroke to date. Surgery has been noted to be an effective stroke-prophylaxis option for the ischemic type and maybe even for the hemorrhagic type [9].

## 4. Conclusion

Moyamoya disease is an unusual cause of stroke in a young adult. Early diagnosis of this rare disease can be complicated by psychiatric manifestation and anchoring bias, like in our case. The clinician should consider this rare condition while evaluating a young patient with stroke in appropriate clinical settings.
